# Altered intrinsic brain activity and functional connectivity in COVID-19 hospitalized patients at 6-month follow-up

**DOI:** 10.1186/s12879-023-08331-8

**Published:** 2023-08-08

**Authors:** Ruili Li, Guangxue Liu, Xiaodong Zhang, Miao Zhang, Jie Lu, Hongjun Li

**Affiliations:** 1 Department of Radiology and Nuclear Medicine, Xuanwu Hospital, Capital Medical University, No.45 Changchun Street, Xicheng District, Beijing, 100053 China; 2 Department of Radiology, Beijing Youan Hospital, Capital Medical University, No.8 Xi Tou Tiao Youanmen Wai, Fengtai District, Beijing, 100069 China; 3 Beijing Key Laboratory of Magnetic Resonance Imaging and Brain Informatics, Beijing, 100053 China; 4 Department of Natural Medicines, School of Pharmaceutical Sciences, Peking University Health Science Center, Beijing, 100191 China; 5 Department of Radiology, Tianjin First Central Hospital, Nankai University, Tianjin, 300192 China

**Keywords:** COVID-19, Brain function, Amplitude of low-frequency fluctuation, Functional connectivity, MRI

## Abstract

**Background:**

Although most patients can recover from SARS-CoV-2 infection during the short-term, the long-term effects of COVID-19 on the brain remain explored. Functional MRI (fMRI) could potentially elucidate or otherwise contribute to the investigation of the long COVID syndrome. A lower fMRI response would be translated into decreased brain activity or delayed signal transferring reflecting decreased connectivity. This research aimed to investigate the long-term alterations in the local (regional) brain activity and remote (interregional) functional connection in recovered COVID-19.

**Methods:**

Thirty-five previously hospitalized COVID-19 patients underwent 3D T_1_weighed imaging and resting-state fMRI at 6-month follow-up, and 36 demographic-matched healthy controls (HCs) were recruited accordingly. The amplitude of low-frequency fluctuation (ALFF) and seed-based functional connectivity (FC) was used to assess the regional intrinsic brain activity and the influence of regional disturbances on FC with other brain regions. Spearman correlation analyses were performed to evaluate the association between brain function changes and clinical variables.

**Results:**

The incidence of neurosymptoms (6/35, 17.14%) decreased significantly at 6-month follow-up, compared with COVID-19 hospitalization stage (21/35, 60%). Compared with HCs, recovered COVID-19 exhibited higher ALFF in right precuneus, middle temporal gyrus, middle and inferior occipital gyrus, lower ALFF in right middle frontal gyrus and bilateral inferior temporal gyrus. Furthermore, setting seven abnormal activity regions as seeds, we found increased FC between right middle occipital gyrus and left inferior occipital gyrus, and reduced FC between right inferior occipital gyrus and right inferior temporal gyrus/bilateral fusiform gyrus, and between right middle frontal gyrus and right middle frontal gyrus/ supplementary motor cortex/ precuneus. Additionally, abnormal ALFF and FC were associated with clinical variables.

**Conclusions:**

COVID-19 related neurological symptoms can self heal over time. Recovered COVID-19 presented functional alterations in right frontal, temporal and occipital lobe at 6-month follow-up. Most regional disturbances in ALFF were related to the weakening of short-range regional interactions in the same brain function.

**Supplementary Information:**

The online version contains supplementary material available at 10.1186/s12879-023-08331-8.

## Introduction

Coronavirus disease 2019 (COVID-19) is a nascent illness caused by the severe acute respiratory syndrome coronavirus-2 (SARS-CoV-2). Due to high infectivity, it soon broke out worldwide and is still ongoing in some areas. In addition to the respiratory system, growing evidence indicates that the liver, kidneys, heart, and brain can also be involved in COVID-19, especially in patients initially reported [1–6]. The most common neurological symptoms included olfactory and gustatory dysfunctions, dizziness, headache, and myalgia. The complications of the central nervous system (CNS), such as encephalitis, encephalopathy, cerebrovascular events, cerebral venous embolism, demyelination, neurodegeneration, and neuroinflammatory syndromes, have also been reported [3, 7–11].

The SARS-CoV-2 RNA could be detected in the cerebral-spinal fluid of COVID-19 patients [7]. Moreover, the definite invasion of the virus in the CNS has been reported in animal research [12], where the viral load of SARS-CoV-2 in the brain of mice is much higher than that in the lungs. There is growing evidence that SARS-CoV-2 has potential neurotropism, just like SARS-CoV [13]. However, it remains unsolved how SARS-CoV-2 affects the human CNS. The direct infection of the virus through transcribrial, hematological, and neuronal retrograde dissemination pathways is an underlying possible pathophysiological mechanism. Other possible mechanisms are hyperimmune-related reactions, including virus-induced hyperinflammatory and hypercoagulable states, and immune-mediated processes [4, 14]. In addition to the above-mentioned neurological symptoms, COVID-19 survivors might have mental problems, including fatigue, poor sleep, anxiety, depression, and post-traumatic stress disorder [15].

MRI can intuitively monitor cerebral structures and function changes non-invasively. Previous neuroimaging studies focused on neurological alterations during the acute infection stage of COVID-19, and a few studies try to explore the brain changes of recovered patients. A cerebral microstructures study showed that volumetric and micro-structural abnormalities mainly involved in the central olfactory cortices and partial white matter in the right hemisphere in recovered COVID-19 patients at 3-month follow-up [16]. Another 1.5-month follow-up study found that olfactory bulb volume and white matter integrity of olfactory regions decreased in COVID-19 patients with anosmia. The results indicated that olfactory bulb injury might be the critical COVID-19-related olfactory dysfunction [17]. Yang et al. [18] demonstrated that 3-month follow-up recovered COVID-19 patients presented widespread white matter damage and abnormal white matter structure network in superior occipital gyrus. Qin et al. [19] found that 3 months after recovery, patients with severe COVID had more decreased cortical thickness/ cerebral blood flow, and more severe white matter damage (mainly in the frontal and limbic systems) than mild ones.

Resting-state fMRI (rs-fMRI) has been widely used in brain mapping to characterize outcomes following encephalitis or the long-term effects of neuropsychiatric diseases on the brain, to shed light on the brain function and its neurobiological mechanisms, and to investigate the association between findings from fMRI and clinical variables [20, 21]. The amplitude of low-frequency fluctuation (ALFF) can measure the changes in the rs-fMRI signals, which could reflect the regional brain activity. Functional connectivity (FC) has been widely used to image the network-level brain function in multiple diseases. To date, the effects of COVID-19 on the brain and the underlying neuropathological mechanisms are ambiguous, and no research has been reported to investigate both intrinsic regional activity and network-level brain function of COVID-19 survivors. This research aimed to characterize the neural activity during rest using ALFF in recovered COVID-19 patients, and further explore whether the regional disturbances affect FC in the local and long-range networks, and assess the associations between clinical characteristics and ALFF values, FC strength of abnormal regional activity.

## Materials and methods

**Fig. 1:**
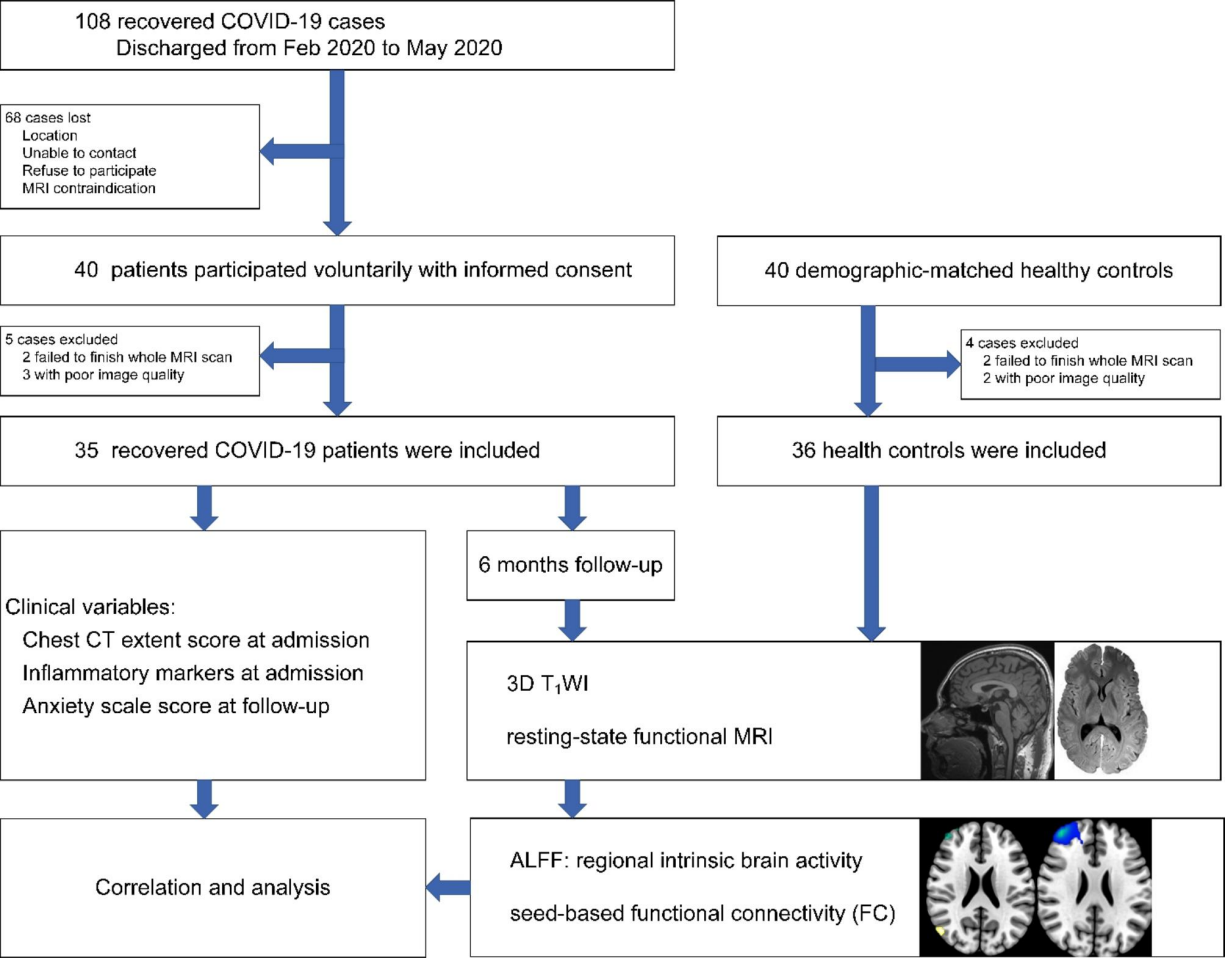
Flow chart of the experimental design

The flow chart of the experimental design is shown in Fig. 1.

### Participants

The Ethics Committee of Beijing Youan Hospital, Capital Medical University, has approved this study. Each participant was informed about the purpose of this research, and written informed consent was acquired. The research has been carried out in accordance with the Declaration of Helsinki of the World Medical Association revised in 2013 for experiments involving humans. A total of 40 recovered COVID-19 patients were discharged from February 2020 to May 2020, and 40 age, sex, and educational-level matched non-COVID-19 healthy controls (HCs) were recruited. The criteria for diagnosis and discharge of COVID-19 patients conformed to published guidelines from the National Health Commission of P.R.China [22]. All participants had a similar experience with the outbreak of COVID-19. The clinical characteristics of patients were extracted from the electronic medical records, including demographical, clinical symptoms, chest CT extent score, and laboratory data at admission. The CT score was evaluated independently for each of the five lobes, and each lobe was scored based on the following criteria [23]: 0, 0%; 1, < 25%; 2, 25 – 50%; 3, 50 – 75%; 4, > 75%. The total extent score was the summation of the five lobes score (range 0–20). The laboratory indicators included C-reactive protein (CRP), alanine aminotransferase (ALT), aspartate aminotransferase (AST), albumin (ALB), globulin (GLOB), albumin/ globulin ratio (A/G ratio), glomerular filtration rate (eGFR), creatine kinase (CK), white blood cell (WBC), lymphocytes (LYM), percentage of LYM (%LYM), neutrophil (NEUT), percentage of NEUT (%NEUT), and procalcitonin (PCT). The neurological symptoms during the acute infection stage and follow-up were collected according to each patient’s self-reports, including headache, myalgia, smell loss, taste loss, fatigue, vision loss, hearing loss, and anxiety. Meanwhile, the recovered COVID-19 patients also completed the self-rating anxiety scale to evaluate their anxiety levels at 6 month follow-up.

### Neuroimaging

#### Protocol

The patients underwent head MRI at 6-month follow-up. MR images were acquired using a 3.0-T MR scanner (Tim-Trio, Siemens, Erlangen, Germany) with a 32-channel phased-array head coil. First, high-resolution T_1_-structural images were acquired using magnetization-prepared rapid gradient echo sequences: repetition time (TR): 1,900 ms; echo time (TE): 2.52 ms; inversion time (TI): 900 ms; flip angle: 9^°^; field of view (FOV): 250 × 250 mm^2^; matrix size: 256 × 246; number of slices: 176; slice thickness: 1 mm; voxel size: 1 × 0.977 × 0.977 mm^3^. Then, the rs-fMRI was acquired using echo-planar imaging (EPI) sequence: TR: 2000 ms; TE: 30 ms; FOV: 224 × 224 mm^2^; matrix: 64 × 64; slice thickness: 3.5 mm; flip angle: 90^°^; voxel size: 3.5 × 3.5 × 4.2 mm^3^; time points: 240. Foam pads were equipped to minimize head movement, and earplugs were utilized to reduce noise. During scanning, all subjects were instructed to stay still, close eyes, think of nothing in particular, and not fall asleep. Five patients and four HCs were excluded due to failure to finish the whole MRI scan or poor image quality. Finally, imaging data of 35 recovered COVID-19 patients and 36 HCs were presented for analysis.

#### Image processing

The rs-fMRI data preprocessing was performed using statistical parametric mapping (SPM12, http://wwwfil.ion.ucl.ac.uk/spm). The first ten time points were discarded to diminish non-equilibrium effects in the fMRI signal. For each subject, the remaining 230 time points were used for slice-timing correction, realigned with the first image in the first series, and then unwrapped to correct susceptibility-by-motion interactions. The time courses of head movements were acquired by estimating the translation in every direction and the rotation in angular movement along every axis for each of the 230 consecutive volumes. None of the participants exhibited head motion of displacement > 1.0 mm or angular motion > 1^°^ at each axis. Nuisance covariates were regressed, including head movement parameters, overall mean signal intensity, cerebrospinal fluid, and white matter signal intensity. The realigned images were spatially normalized into Montreal Neurological Institute (MNI) space, resampled to 3 × 3 × 3 mm^3^, and smoothed with a full-width-at-half-maximum Gaussian kernel of 8 mm.

#### ALFF calculation

ALFF can be utilized to quantify the spontaneous fluctuation of fMRI signal intensity, and has been considered a reliable biomarker for neurological diseases [24]. As previously mentioned, ALFF analysis was carried out using the fMRI data analysis toolkit [25]. First, the fast Fourier transform was utilized to extract the power spectrum after detrending the time series. Then, the average ALFF within each voxel was calculated across the frequency band 0.01 – 0.08 Hz. Finally, the ALFF of every voxel was divided by the overall mean ALFF value of the subject to normalize data across subjects.

#### Functional connectivity analysis

Set brain regions with significant ALFF differences as seeds for FC analysis. After band-pass filtering (0.01 – 0.08 Hz) and linear trend elimination, a reference time series of each seed was extracted by averaging the rs-fMRI time series of voxels in each seed. Pearson’s correlation coefficients were calculated voxel by voxel between the time series in each region of interest (ROI) and the filtered time series in the rest of brain [26]. Fisher’s *r*-to-*z* transformation is used to convert the correlation coefficients in each voxel into *z* scores to improve normality.

### Statistical analysis

Differences in demographic information between recovered COVID-19 and HCs were examined with IBM SPSS 20.0 (IBM Inc. Armonk, NY, USA). The Shapiro-Wilk test was performed to assess the normal distribution of variables. Mann-Whitney U test, *χ*^2^ test, or Fisher’s exact test were used to compare the differences in age, education level, sex distribution, and complication between case and control groups, appropriately. The significance threshold was set to 0.05, two-tailed. The differences in ALFF and FC between recovered COVID-19 and HCs were performed with a two-sample *t-test* using SPM12. The topological false discover rate (FDR) was used for multiple comparisons correction with the significance threshold 0.05. In the recovered COVID-19 group, Spearman correlation analyses were performed to evaluate the relationship between brain function changes (altered ALFF and FC) and clinical variables (chest CT extent score, laboratory data, and anxiety scale score).

## Results

### Demographic and clinical characteristics

Thirty-five recovered COVID-19 patients (moderate: 27, 77.14%; severe: 8, 22.86%) and 36 demographically matched HCs were included. During COVID-19 hospitalization stage, 21/35 (60%) patients had neurological symptoms, including fatigue (13, 37.14%), myalgia (9, 25.71%), taste loss (6, 17.14%), smell loss (4, 11.43%), headache (4, 11.43%), vision loss (1, 2.86%), hearing loss (1, 2.86%), and anxiety (1, 2.86%). At the 6-month follow-up, neurological symptoms improved in most patients, and 6/35 (17.14%) patients still had neurological symptoms, including fatigue (3, 8.57%), taste loss (1, 2.86%), vision loss, hearing loss (1, 2.86%), and anxiety (1, 2.86%) (see Table 1).

**Table 1:** Summary of demographic information and clinical characteristics

Demographic and clinical data	Recovered COVID-19 (*n* = 35)	Healthy Controls (*n* = 36)	*P*-value
Age, years	43 (35, 58)	46 (35, 56.5)	0.950^a^
Sex, male/female	17/18	19/17	0.723^b^
Education, years	15 (10, 18)	16 (11.25, 16)	0.949^a^
**Underlying diseases**, ***n*(%)**			
Hypertension	3 (8.57%)	3 (8.33%)	1.000^c^
Diabetes	3 (8.57%)	2 (5.56%)	0.674^c^
Coronary heart disease	1 (2.86%)	1 (2.78%)	1.000^c^
**Clinical type**, ***n*(%)**			
Moderate type	27 (77.14%)	-	-
Severe type	8 (22.86%)	-	-
**CT score of lung involvement at admission**, Median (IQR)	4 (2, 7)	-	-
**Time between discharge and follow-up** (months)	6.49 ± 1.10	-	-
**Nervous system symptoms at admission**			
Fatigue	13 (37.14%)	-	-
Myalgia	9 (25.71%)	-	-
Taste loss	6 (17.14%)	-	-
Smell loss	4 (11.43%)	-	-
Headache	4 (11.43%)	-	-
Vision loss	1 (2.86%)	-	-
Hearing loss	1 (2.86%)	-	-
Anxiety	1 (2.86%)	-	-
**Nervous system symptoms at 6-month follow-up**			
Fatigue	3 (8.57%)	-	-
Taste loss	1 (2.86%)	-	-
Vision loss	1 (2.86%)	-	-
Hearing loss	1 (2.86%)	-	-
Anxiety	1 (2.86%)	-	-
**Laboratory tests at admission**			
C-reactive protein	20.00 (7.10, 51.1)	-	-
White blood cell, 10^9^/L	5.35 ± 1.72	-	-
Lymphocyte, 10^9^/L	1.16 ± 0.49	-	-
Lymphocyte percent	23.98 ± 11.84	-	-
Neutrophil	2.94 (2.02, 4.24)	-	-
Neutrophil percent	65.34 ± 13.00	-	-
Albumin	35.59 ± 4.23	-	-
Globulin	35.99 ± 4.12	-	-
Albumin/ globulin ratio	1.01 ± 0.20	-	-

Note: ^a^ Mann-Whitney U test; ^b^*χ*^2^ test; ^c^ Fisher’s exact test

### ALFF group differences

**Fig. 2:**
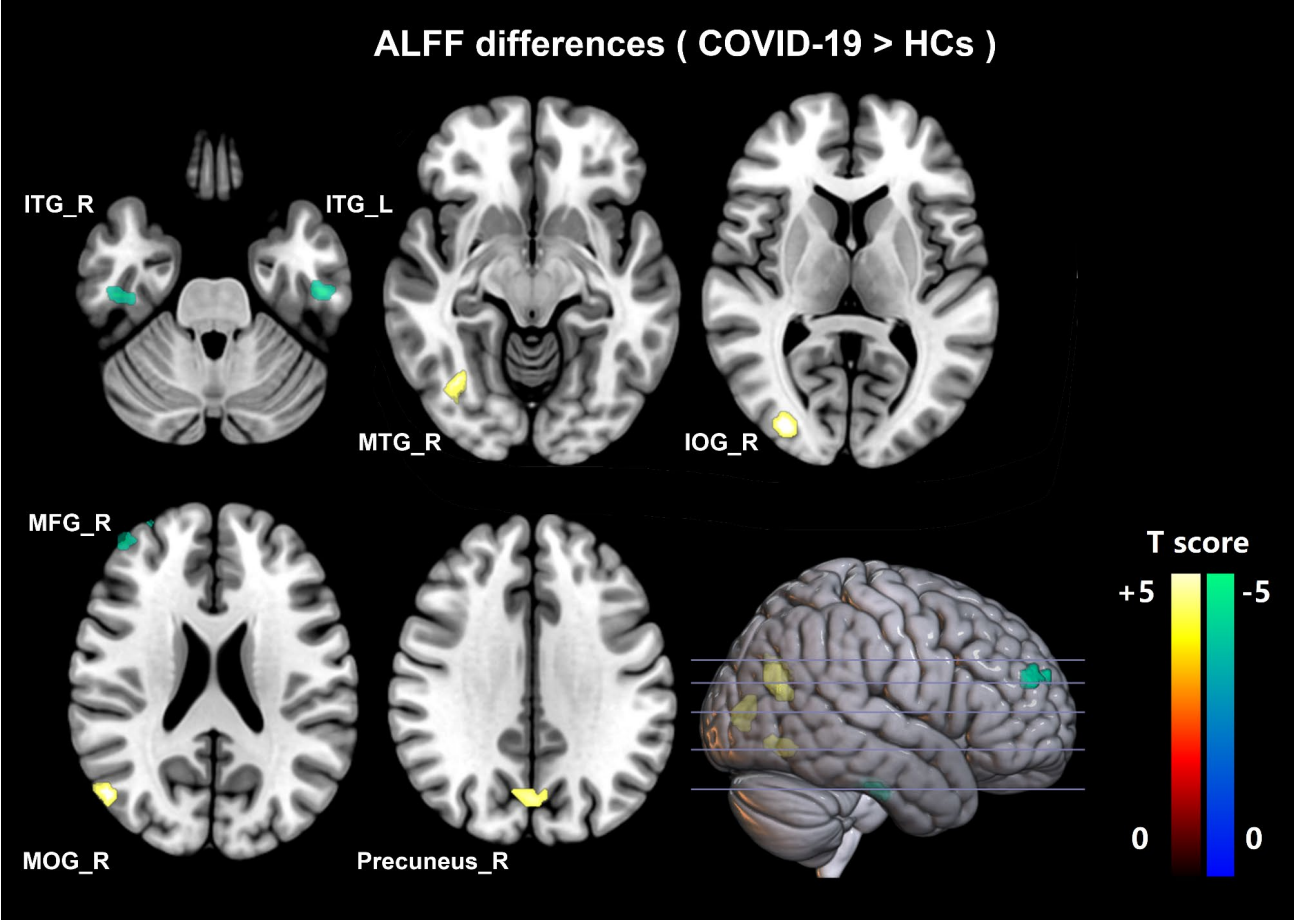
**Regional ALFF differences between recovered COVID-19 patients and healthy controls**. Yellow regions show increased ALFF (hyperactivity), and green regions show decreased ALFF (hypoactivity) in the recovered COVID-19 patients. The difference was calculated by two-tailed two-sample *t-test*, and the results were FDR-corrected (*P* < 0.05). ALFF, amplitude of low-frequency fluctuations; HCs, healthy controls; ITG_R, right inferior temporal gyrus (visual processing); ITG_L, left inferior temporal gyrus (visual processing); MTG_R, right middle temporal gyrus (memory, attention, auditory, visual processing); IOG_R, right inferior occipital gyrus (visual processing); MFG_R, right middle frontal gyrus (executive functioning, working memory, attention); MOG_R, right middle occipital gyrus (visual processing); Precuneus_R, right precuneus (visual information, memory, attention)

A total of seven ALFF clusters with significant differences were identified between recovered COVID-19 and HCs. Four clusters (the right precuneus, middle temporal gyrus, middle occipital gyrus, and inferior occipital gyrus) exhibited increased ALFF (hyperactivity), and three clusters (right middle frontal gyrus, inferior temporal gyrus, and left inferior temporal gyrus) showed decreased ALFF (hypoactivity). All results were topological FDR corrected with *P* < 0.05. (see Table 2; Fig. 2, and Figure S1).

**Table 2:** Amplitude of low-frequency fluctuation (ALFF) differences between recovered COVID-19 and healthy controls (HCs)

ROI	Brain areas	Functions of brain areass [25]	Brodmann area	MNI coordinates (X,Y,Z) mm	Number of voxels	Peak *t-*value^a^
1	right precuneus	visual information memory attention	7	6, -69, 30	54	3.9439
2	right middle temporal gyrus	memory attention auditory visual processing	39	51, -69, 18	53	4.2999
3	right middle occipital gyrus	visual processing	18/19	33, -87, 6	50	4.1681
4	right inferior occipital gyrus	visual processing	-	33, -63, -9	30	4.0042
5	right middle frontal gyrus	executive functioning working memory attention	10	42, 51, 27	34	-4.0024
6	right inferior temporal gyrus	visual processing	20	48, -21, -30	23	-4.3546
7	left inferior temporal gyrus	visual processing	20	-48, -18, -30	34	-4.2425

Note: The differences in ALFF between recovered COVID-19 and HCs were performed with a two-sample *t-test*, and the results were FDR-corrected (*P* < 0.05). ^a^ Positive *t*-value means ALFF_COVID−19_ > ALFF_HC_. Negative *t*-value means ALFF_COVID−19_ < ALFF_HC_.

Brodmann areas of the cerebral cortex are defined by cytoarchitecture, proposed by the German anatomist Korbinian Brodmann in the early 1900’s

MNI coordinates represents the spatial coordinate position of the brain area. It refer to the normalized space defined in SPM12, which is in mm, and the (X, Y, Z) axes are oriented towards (right, anterior, posterior). The origin is at the AC (anterior commissure) point of the template, with the (negative) Y axis also going through the PC (posterior commissure) point of the template

Number of voxels represent the size of brain areas

### Altered interregional functional connectivity

**Fig. 3:**
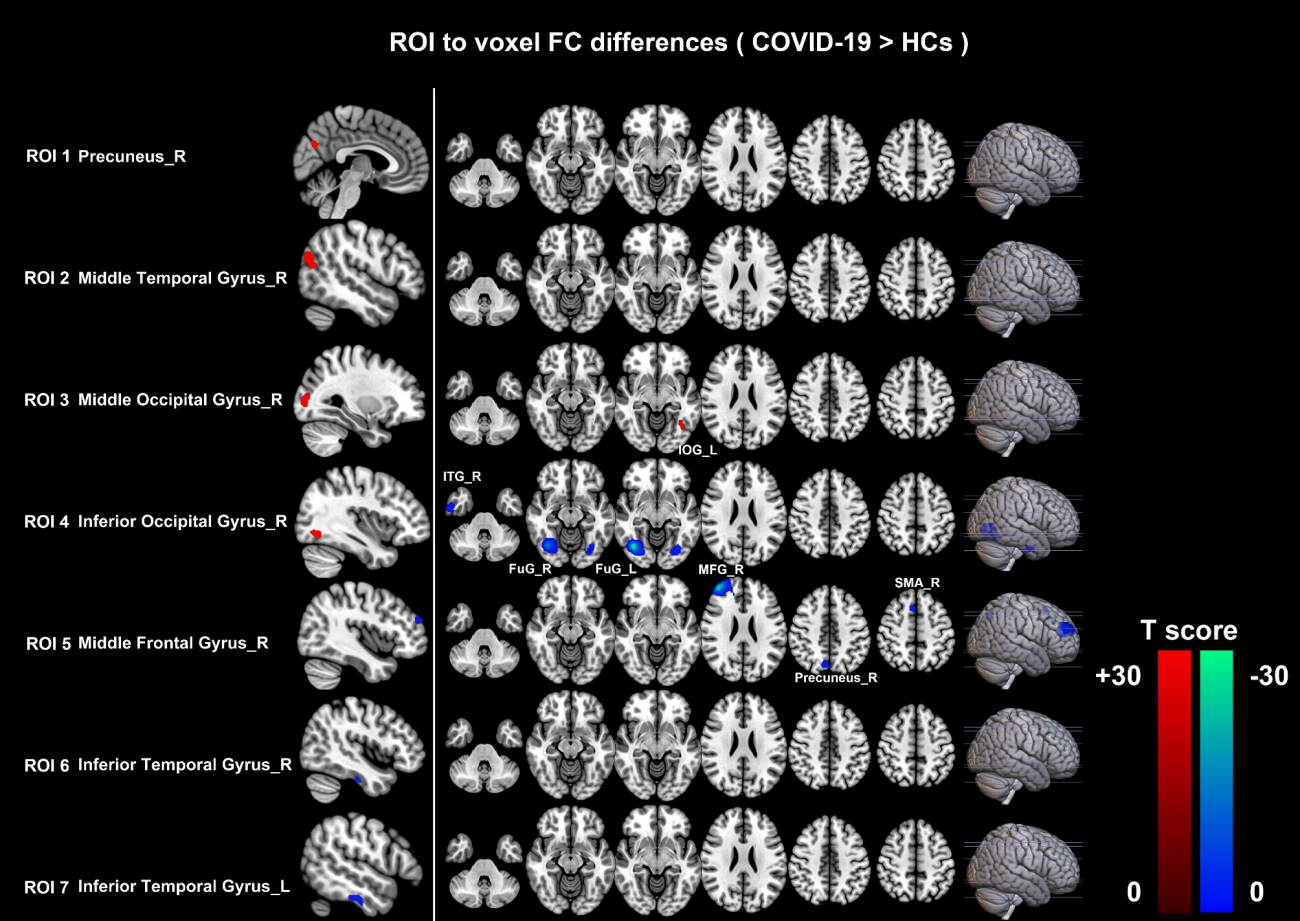
**Abnormalities in the seed-based functional connectivity.** Red regions showed increased FC values, and Blue regions showed decreased FC values in the recovered COVID-19 patients. The difference was calculated by two-tailed two-sample *t-test*, and the results were FDR-corrected (P < 0.05). FC, functional connectivity; HCs, healthy controls; ROI, region of interest; R, right; L, left; IOG_L, left inferior occipital gyrus (visual processing); ITG_R, right inferior temporal gyrus (visual processing); FuG_R, right fusiform gyrus (visual processing); FuG_L, left fusiform gyrus (visual processing); MFG_R, right middle frontal gyrus (executive functioning, working memory, attention); SMA_R, right supplementary motor area (language, motor); Precuneus_R, right precuneus (visual information, memory, attention)

In our research, seven difference clusters were respectively defined as the seeds to investigate the effects of abnormal intrinsic activity on FC. Increased ALFF in the right precuneus (ROI 1) and middle temporal gyrus (ROI 2), and decreased ALFF in the bilateral inferior temporal gyrus (ROI 6,7) have not produced aberrant FC profiles. Setting right middle occipital gyrus (ROI 3) with higher ALFF as seed, recovered COVID-19 patients showed higher FC between ROI 3 and left inferior occipital gyrus. Lower FC could be observed between the seed (right inferior occipital gyrus with higher ALFF, ROI 4) and the right fusiform gyrus, inferior temporal gyrus, and left fusiform gyrus. Moreover, lower FC can also be detected between the seed (right middle frontal gyrus with lower ALFF, ROI 5) and the right frontal middle gyrus, supplementary motor area, and precuneus. All results were topological FDR corrected, *P* < 0.05. (see Table 3; Fig. 3, and Figure S2)

**Table 3:** Seed-based functional connectivity (FC) differences between recovered COVID-19 and healthy controls (HCs)

ROI	Seed	Connected areas	Functions of connected areas [25]	MNI coordinates (X,Y,Z) mm	Number of voxels	Peak *t*-value^a^
3	right middle occipital gyrus	left inferior occipital gyrus	visual processing	-39, -57, -9	26	3.8931
4	right inferior occipital gyrus	right fusiform gyrus	visual processing	39, -69, -9	341	-21.986
		right inferior temporal gyrus	visual processing	57, -9, -33	55	-4.5721
		left fusiform gyrus	visual processing	-30, -75, -9	67	-4.7662
5	right middle frontal gyrus	right middle frontal gyrus	executive functioning working memory attention	39, 51, 24	355	-26.164
		right supplementary motor area	language motor	6, 18, 54	58	-4.1750
		right precuneus	visual information memory attention	6, -72, 45	48	-3.9974

Note: Set brain regions with significant ALFF differences as seeds for FC analysis. Altered ALFF in the right precuneus (ROI 1), middle temporal gyrus (ROI 2) and bilateral inferior temporal gyrus (ROI 6,7) have not produced aberrant FC profiles. The differences in FC between recovered COVID-19 and HCs were performed with a two-sample *t-test*, and the results were FDR-corrected (*P* < 0.05). ^a^ Positive *t*-value means FC_COVID−19_ > FC_HC_. Negative *t*-value means FC_COVID−19_ < FC_HC_. The meaning of MNI coordinates and Number of voxels are the same as Table 2

### Associations between brain function alterations and clinical characteristics

**Fig. 4:**
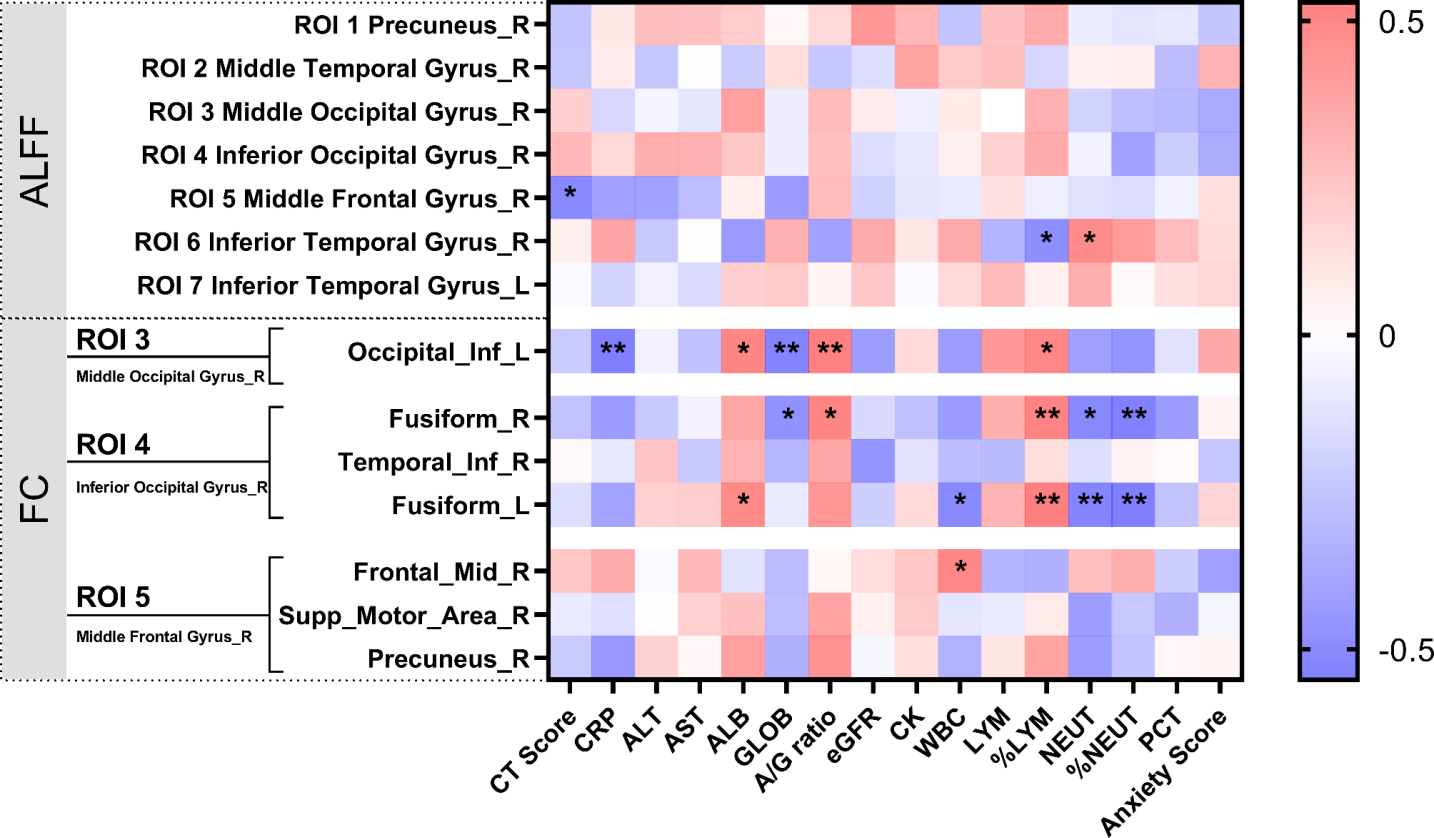
**Spearman correlations between brain function alterations and clinical indicators.** Different colors in the figure showed the Spearman correlation coefficient. ALFF, amplitude of low-frequency fluctuations; FC, functional connectivity; ROI, region of interest; R, right; L, left; CRP, C-reactive protein; ALT, alanine aminotransferase, AST, aspartate aminotransferase; ALB, albumin; GLOB, globulin; A/ G ratio, albumin/ globulin ratio; eGFR, glomerular filtration rate; CK, creatine kinase; WBC, white blood cell; LYM, lymphocytes; %LYM, percentage of LYM; NEUT, neutrophil; %NEUT, percentage of NEUT; PCT, procalcitonin. ^*^, Significant level *P* < 0.05; ^**^, Significant level *P* < 0.01

In the right middle frontal gyrus, ALFF is negatively correlated with CT score of lung involvement (*r* = -0.411, *P* = 0.014). In the right inferior temporal gyrus, ALFF is negatively correlated with %LYM (*r* = -0.363, *P* = 0.032), and positively correlated with NEUT (*r* = 0.369, *P* = 0.029) (see Table S1, Fig. 4). The FC between right middle occipital gyrus (ROI 3) and left inferior occipital gyrus is negatively correlated with CRP (*r* = -0.519, *P* = 0.001), GLOB (*r* = -0.455, *P* = 0.006), and positively correlated with ALB (*r* = 0.425, *P* = 0.011), A/ G ratio (*r* = 0.529, *P* = 0.001), and %LYM (*r* = 0.399, *P* = 0.018). Between right inferior occipital gyrus (ROI 4) and right fusiform gyrus, the FC is negatively correlated with GLOB (*r* = -0.345, *P* = 0.043), NEUT (*r* = -0.398, *P* = 0.018), %NEUT (*r* = -0.503, *P* = 0.002), and positively correlated with A/ G ratio (*r* = 0.417, *P* = 0.013), and %LYM (*r* = 0.438, *P* = 0.008). Moreover, lower FC between right inferior occipital gyrus (ROI 4) and left fusiform gyrus means higher WBC (*r* = -0.398, *P* = 0.018), NEUT (*r* = -0.435, *P* = 0.009), %NEUT (*r* = -0.549, *P* = 0.001), and lower ALB (*r* = 0.376, *P* = 0.026), and %LYM (*r* = 0.516, *P* = 0.002). Finally, the FC between the right middle frontal gyrus (ROI 5) and right frontal middle is positively correlated with WBC (*r* = 0.409, *P* = 0.015) (see Table S2, Fig. 4).

## Discussion

Though the pneumonia was completely absorbed and the neurological symptoms have improved in most patients at the 6-month follow-up, 17.14% of patients still had neurological symptoms, including fatigue, taste loss, vision loss, hearing loss, and anxiety. By using ALFF and FC, our study analyzed the effect of COVID-19 on brain function in recovered patients in 2020. At the 6-month follow-up, the recovered patients exhibited ALFF alterations mainly in the right cerebral hemisphere, including the right middle temporal gyrus, precuneus, middle/ inferior occipital gyrus, middle frontal gyrus, and bilateral inferior temporal gyrus. Furthermore, setting the abnormal activity regions as seeds, reduced FC was also shown in the right hemisphere (between the right inferior occipital gyrus and right inferior temporal gyrus/ bilateral fusiform gyrus; between the right middle frontal gyrus and right middle frontal gyrus/ supplementary motor cortex/ precuneus). An interesting finding was that regional disturbances in ALFF were predominantly associated with weakened short-range interactions with regions in similar functions, including memory, attention and visual processing [27]. Additionally, abnormal ALFF and FC values were related to clinical variables.

It was reported in early research that COVID-19 patients can present various neurological symptoms and complications [3, 7–11]. In the acute infection stage, the abnormal neuroimaging findings mainly occurred in frontal/ temporal/ parietal/ occipital lobes [28–31]. A COVID-19 patient presented symmetric bilateral hypodensity in the subcortical white matter of posterior frontal and temporo-parieto-occipital on the 25th day of admission. On the 56th day, the hypodensity reduced significantly [28]. COVID-19 patients presented hyperintensity in bilateral frontal, parietal and occipital cortex in T_2_-weighted images, and the lesions demonstrated possible spontaneous reversibility [30] or absorption after corticosteroid treatment [29]. A COVID-19 patient with anosmia presented hyperintensity in the right orbital prefrontal cortex next to the olfactory bulb, and the lesion disappeared after one month [32]. The above findings indicate the self-recovery of the brain.

Recovered COVID-19 patients also displayed brain micro changes. A 3-month follow-up study of recovered COVID-19 patients without neurological symptoms at initial infection showed that cerebral blood flow in the severe group was significantly reduced across the gray matter cortex (mainly in bilateral frontal and temporal cortex) compared with HCs [19]. In our research, at 6-month follow-up, the recovered COVID-19 patients exhibited decreased ALFF in the right frontal lobe and bilateral temporal lobes and increased ALFF mainly in the right parietal and occipital lobe compared to HCs. These findings indicate that the frontal and temporal lobes alterations presented in not only the acute infection period but also the recovery period. Moreover, we found that the ALFF value of the right middle frontal gyrus was negatively correlated with the CT scores of lung involvement, which suggested that the more severe the lung lesion, the lower the spontaneous activity of frontal lobe. The severity of lung lesions is associated with hypoxia, and frontal lobe might be vulnerable to hypoxia in COVID-19, which is in line with a previous study [33]. Clinically, hypoxia can cause the dysfunction of the blood-brain barrier and anaerobic metabolism in neuronal mitochondria, and lead to a decrease in cortical blood flow and spontaneous brain activity [34]. Neuropathologic characteristics of autopsied COVID-19 also exhibited various hypoxia damage in the brain, such as the enlargement of the perivascular space with abundant cells, and there were no specific signs of meningitis or encephalitis [35, 36]. Increased ALFF in parietal and occipital lobes might reflect rapid recovery, reorganization, or compensation of brain function.

ALFF can reflect the alteration of local brain activity by measuring the fluctuation of blood oxygen level dependent (BOLD) signal. Factors leading to abnormal BOLD signals, such as glucose metabolism and cerebral blood flow, are considered as the functional basis of abnormal ALFF. Decreased ALFF in the right frontal lobe and bilateral temporal lobes in recovered COVID-19 indicate the decrease of metabolic activity or cerebral blood flow in these regions. A series of 18 F-FDG-PET cases demonstrated the hypometabolism predominant in the prefrontal and orbitofrontal cortexes. A COVID-19 patient with anosmia presented metabolic activity reduced in the orbitofrontal cortex [37]. Recovered COVID-19 patients with functional impairments (dyspnea, ageusia, anosmia, insomnia, pain, memory impairment) presented hypometabolism in frontal and right temporal lobes for at least three weeks [38]. COVID-19 patients with cognitive impairment showed frontal hypometabolism [39, 40]. Moreover, frontal hypometabolism still existed at a 6-month follow-up [40]. Bilateral frontotemporal hypoperfusion was also noted in recovered patients for 10 months after discharge, however, the range of hypoperfusion was reduced compared with the 3-month follow-up findings [41].

In terms of the FC changes in the current study, it was noteworthy that regional alterations in ALFF predominantly resulted in the deduction of FC between adjacent, short-range, or within the same functional regions, which are mainly responsible for executive functioning, memory, attention, language, motor, and visual processing [27]. The lack of detailed neurocognitive tests of rehabilitation patients is a defect of our study, which makes it impossible for us to conduct the assessment of cognitive deficits and the correlation between altered ALFF, FC, and neurocognitive tests. However, it is reported that most patients had impairments in attention, processing speed, long-term verbal and visuospatial memory at 5-month follow-up [42]. We found that the higher inflammatory related clinical variables (CRP, PCT, WBC, NEUT, and %NEUT), and the lower LYM, %LYM, ALB, and A/G ratio means the weaker FC of these brain regions. It indicates that the worse the status of patients at admission, the lower the FC in recovery. The inflammatory variables are not only correlated with the severity of the COVID-19, but also closely related to the evolution of the disease, which was reported in our previous research [43]. Inflammatory variables at admission may predict brain function changes at a half-year follow-up.

The underlying pathological mechanisms of COVID-19-related brain alteration are still ambiguous. The persistent frontotemporal lobe involvement might provide insight into the early route of SARS-CoV-2 brain invasion and pathogenesis. Anosmia is a common clinical symptom of COVID-19 [44]. The loss of olfaction, secondary to nasal congestion or changes in conduction pathways, are known sequela of rhinovirus infection [45]. However, there is less evidence of the association between anosmia of COVID-19 and nasal congestion [46]. In our study, none of the five patients with gustatory impairment and four with anosmia had nasal congestion, which suggests that sensory nerve loss in frontal cortex might be the underlying cause of olfactory dysfunction. Frontotemporal lobe involvement might be attributed to hypoxia, and the influence of stressful events or other processes, such as immune response [47]. SARS-CoV-2 might act on angiotensin-converting enzyme 2 (ACE2) receptors, which could lead to dysregulation of the renin angiotensin system, microcirculation impairment, and blood flow regulation dysfunction [48], and the frontal cortex is one of the brain regions which frequently expresses ACE2 [16, 49].

It was interesting to notice that abnormal spontaneous brain activity and FC were mainly in the right hemisphere in our research, which was as same as a previous structural study [16, 50]. This asymmetrical right-lateralized phenomenon of recovered COVID-19 patients was not fully understood. There may be putative several reasons for this. On one hand, the acute infection stage mainly involves the right and bilateral cerebral hemispheres. The first reported case of meningitis/encephalitis associated with SARS-CoV-2 had hyperintensity in the right mesial temporal lobe, hippocampus and periventricular [7]. A COVID-19 patient with anosmia presented alterations in the right orbital prefrontal cortex [32]. Other previous studies often reported bilateral abnormalities of CNS in the acute SARS-CoV-2 infection stage [28–30]. We speculated that the right hemisphere might be more vulnerable to SARS-CoV-2 infection or more severe during acute infection, and the recovery rate in the right hemisphere was slower than the left. On the other hand, researches on other diseases also present a right-lateralized phenomenon, such as posttraumatic stress disorder [50–52], paranoid schizophrenia [53], bipolar disorder [54], and primary insomnia [55]. The long-lasting pandemic of COVID-19, impact on patients’ mental health is also long-term, so another speculation might be related to the neurological and neuropsychiatric consequences (psychological effects, stress, anxiety, fear, depression, insomnia, etc.) of COVID-19. In addition, the right hemisphere plays a dominant role in some functions, including visual working memory[56], attention (pain processing) [57], subliminal face processing[58], maintaining phasic alerting capabilities [59], spatial and verbal domains [60], verbal episodic memory retrieval [61], and our significant brain regions just mainly participates in memory, attention, and visual processing. However, the real mechanism of right-lateralized phenomenon in our patient sample is still unclear. In the future, we hope to recruit more patients to verify whether a lateralized effect and to explore the real mechanism.

The current study has several limitations. First, more clinical attention focused on viral infection and lung inflammation rather than the slight neurological manifestations at the acute stage. The lack of MR neuroimaging data at admission limits the evaluation of brain function alterations. Secondly, single-center and limited sample size might weaken the statistical power of this research. Finally, all the recruited COVID-19 patients were moderate and severe types, which may prevent research data from reflecting the full spectrum of COVID-19 patients (mild and critical types).

## Conclusion

In conclusion, the incidence of neurosymptoms at half-year follow-up declined significantly compared with the COVID-19 hospitalization stage, indicating self-recovery of nervous system over time. ALFF and FC alterations in the right frontal, temporal, and occipital lobes remained in COVID-19 survivors after a half-year recovery. Most regional disturbances in ALFF were associated with weakened short-range interactions with regions in the same function. These findings provide new evidence for the effect of COVID-19 on the brain.

### Electronic supplementary material

Below is the link to the electronic supplementary material.

Supplementary Material 1

## Data Availability

The data that support the findings of this study are available from the corresponding author, upon reasonable request.

## Abbreviations

COVID-19: Coronavirus disease 2019
SARS-CoV-2: severe acute respiratory syndrome coronavirus-2
fMRI: Functional magnetic resonance imaging
rs-fMRI: Resting-state fMRI
HCs: Healthy controls
ALFF: Amplitude of low-frequency fluctuation
FC: Functional connectivity
CNS: Central nervous system
CT: Computed tomography
ROI: Region of interest
FDR: False discovery rate
BOLD: Blood oxygen level dependent
CRP: C-reactive protein
ALT: Alanine aminotransferase
AST: Aspartate aminotransferase
ALB: Albumin
GLOB: Globulin
A/G ratio: Albumin/ globulin ratio
eGFR: Estimated glomerular filtration rate
CK: Creatine kinase
WBC: White blood cell
LYM: Lymphocytes
%LYM: Percentage of LYM
NEUT: Neutrophil
%NEUT: Percentage of NEUT
PCT: Procalcitonin
