# Supplementary material for: Altered intrinsic brain activity and functional connectivity in COVID-19 hospitalized patients at 6-month follow-up
Source: BMC Infect Dis. 2023 Aug 8;23:521. doi: 10.1186/s12879-023-08331-8 (PMC10410836; doi:10.1186/s12879-023-08331-8)
Supplement: Supplementary file 1 — Supplementary Material 1 [file 12879_2023_8331_MOESM1_ESM.docx]

**Supplementary Material**

**Table S1** Two-tailed Spearman correlation analysis of ALFF and clinic indicators

|  |  | **ROI 1**  **Precuneus_R** | **ROI 2**  **Middle Temporal Gyrus_R** | **ROI 3**  **Middle Occipital Gyrus_R** | **ROI 4**  **Inferior Occipital Gyrus_R** | **ROI 5**  **Middle Frontal Gyrus_R** | **ROI 6**  **Inferior Temporal Gyrus_R** | **ROI 7**  **Inferior Temporal Gyrus_L** |
| --- | --- | --- | --- | --- | --- | --- | --- | --- |
| CT Score | *r* | -0.154 | -0.142 | 0.113 | 0.182 | **-0.411^*^** | 0.036 | -0.015 |
|  | *P*-value | 0.376 | 0.416 | 0.516 | 0.295 | **0.014** | 0.837 | 0.930 |
| CRP | *r* | 0.049 | 0.042 | -0.097 | 0.086 | -0.270 | 0.243 | -0.108 |
|  | *P*-value | 0.781 | 0.810 | 0.580 | 0.623 | 0.117 | 0.159 | 0.537 |
| ALT | *r* | 0.156 | -0.139 | -0.024 | 0.215 | -0.265 | -0.136 | -0.036 |
|  | *P*-value | 0.372 | 0.427 | 0.891 | 0.215 | 0.124 | 0.435 | 0.837 |
| AST | *r* | 0.148 | 0.006 | -0.059 | 0.202 | -0.168 | 0.006 | -0.088 |
|  | *P*-value | 0.397 | 0.973 | 0.736 | 0.246 | 0.335 | 0.971 | 0.617 |
| ALB | *r* | 0.120 | -0.128 | 0.266 | 0.136 | 0.036 | -0.293 | 0.110 |
|  | *P*-value | 0.493 | 0.462 | 0.123 | 0.437 | 0.836 | 0.088 | 0.530 |
| GLOB | *r* | 0.015 | 0.074 | -0.041 | -0.041 | -0.290 | 0.200 | 0.128 |
|  | *P*-value | 0.931 | 0.673 | 0.816 | 0.816 | 0.092 | 0.250 | 0.463 |
| A/G ratio | *r* | 0.089 | -0.138 | 0.168 | 0.153 | 0.163 | -0.267 | 0.020 |
|  | *P*-value | 0.612 | 0.428 | 0.334 | 0.380 | 0.350 | 0.121 | 0.907 |
| eGFR | *r* | 0.305 | -0.080 | 0.043 | -0.081 | -0.114 | 0.225 | 0.132 |
|  | *P*-value | 0.074 | 0.646 | 0.805 | 0.644 | 0.513 | 0.193 | 0.449 |
| CK | *r* | 0.183 | 0.250 | -0.038 | -0.057 | -0.060 | 0.057 | -0.013 |
|  | *P*-value | 0.293 | 0.147 | 0.830 | 0.744 | 0.734 | 0.744 | 0.940 |
| WBC | *r* | -0.150 | 0.123 | 0.047 | 0.030 | -0.051 | 0.223 | 0.081 |
|  | *P*-value | 0.391 | 0.482 | 0.788 | 0.864 | 0.769 | 0.197 | 0.644 |
| LYM | *r* | 0.147 | 0.153 | <0.001 | 0.106 | 0.070 | -0.196 | 0.171 |
|  | *P*-value | 0.401 | 0.379 | 0.999 | 0.543 | 0.690 | 0.260 | 0.325 |
| %LYM | *r* | 0.218 | -0.095 | 0.201 | 0.221 | -0.040 | **-0.363^*^** | 0.035 |
|  | *P*-value | 0.208 | 0.585 | 0.247 | 0.202 | 0.818 | **0.032** | 0.841 |
| NEUT | *r* | -0.044 | 0.035 | -0.109 | -0.031 | -0.071 | **0.369^*^** | 0.209 |
|  | *P*-value | 0.803 | 0.842 | 0.534 | 0.858 | 0.683 | **0.029** | 0.228 |
| %NEUT | *r* | -0.059 | 0.037 | -0.163 | -0.265 | -0.083 | 0.272 | 0.014 |
|  | *P*-value | 0.736 | 0.833 | 0.351 | 0.123 | 0.637 | 0.114 | 0.938 |
| PCT | *r* | -0.055 | -0.171 | -0.179 | -0.123 | -0.031 | 0.164 | 0.070 |
|  | *P*-value | 0.756 | 0.334 | 0.310 | 0.488 | 0.862 | 0.355 | 0.693 |
| Anxiety Score | *r* | -0.148 | 0.192 | -0.235 | -0.228 | 0.071 | 0.078 | 0.093 |
|  | *P*-value | 0.396 | 0.268 | 0.174 | 0.188 | 0.687 | 0.656 | 0.596 |

Note: ALFF, amplitude of low-frequency fluctuation; ROI, region of interest; R, right; L, left; *r*, Spearman correlation coefficient; C-reactive protein, CRP; alanine aminotransferase, ALT; aspartate aminotransferase, AST; albumin, ALB; globulin, GLOB; albumin/ globulin ratio, A/ G ratio; glomerular filtration rate, eGFR; creatine kinase, CK; white blood cell, WBC; lymphocytes, LYM; percentage of LYM, %LYM; neutrophil, NEUT; percentage of NEUT, %NEUT; procalcitonin, PCT. ^*^, Significant level *P* < 0.05; ^**^, Significant level *P* < 0.01.

**Table S2** Two-tailed Spearman correlation analysis of FC and clinic indicators

|  |  | **Occipital_Inf_L** | **Fusiform_R** | **Temporal_**  **Inf_R** | **Fusiform_L** | **Frontal_**  **Mid_R** | **Supp_Motor_Area_R** | **Precuneus_R** |
| --- | --- | --- | --- | --- | --- | --- | --- | --- |
| CT Score | *r* | -0.129 | -0.155 | 0.014 | -0.080 | 0.136 | -0.051 | -0.131 |
|  | *P*-value | 0.460 | 0.375 | 0.937 | 0.650 | 0.434 | 0.772 | 0.455 |
| CRP | *r* | **-0.519^**^** | -0.292 | -0.056 | -0.266 | 0.223 | -0.079 | -0.313 |
|  | *P*-value | **0.001** | 0.089 | 0.748 | 0.122 | 0.198 | 0.650 | 0.067 |
| ALT | *r* | -0.028 | -0.133 | 0.142 | 0.107 | -0.015 | 0.004 | 0.103 |
|  | *P*-value | 0.873 | 0.446 | 0.417 | 0.542 | 0.932 | 0.983 | 0.556 |
| AST | *r* | -0.157 | -0.030 | -0.136 | 0.114 | 0.175 | 0.106 | 0.015 |
|  | *P*-value | 0.367 | 0.866 | 0.435 | 0.513 | 0.315 | 0.546 | 0.930 |
| ALB | *r* | **0.425^*^** | 0.237 | 0.197 | **0.376^*^** | -0.067 | 0.150 | 0.262 |
|  | *P*-value | **0.011** | 0.171 | 0.258 | **0.026** | 0.704 | 0.390 | 0.128 |
| GLOB | *r* | **-0.455^**^** | **-0.345^*^** | -0.189 | -0.049 | -0.174 | -0.168 | -0.211 |
|  | *P*-value | **0.006** | **0.043** | 0.278 | 0.780 | 0.318 | 0.334 | 0.224 |
| A/G ratio | *r* | **0.529^**^** | **0.417^*^** | 0.233 | 0.311 | 0.017 | 0.247 | 0.331 |
|  | *P*-value | **0.001** | **0.013** | 0.179 | 0.069 | 0.925 | 0.153 | 0.052 |
| eGFR | *r* | -0.286 | -0.089 | -0.325 | -0.119 | 0.075 | 0.031 | -0.020 |
|  | *P*-value | 0.096 | 0.611 | 0.057 | 0.495 | 0.669 | 0.859 | 0.909 |
| CK | *r* | 0.087 | -0.160 | -0.068 | 0.087 | 0.135 | 0.126 | 0.071 |
|  | *P*-value | 0.620 | 0.360 | 0.696 | 0.621 | 0.439 | 0.472 | 0.686 |
| WBC | *r* | -0.286 | -0.292 | -0.171 | **-0.398^*^** | **0.409^*^** | -0.062 | -0.208 |
|  | *P*-value | 0.096 | 0.088 | 0.325 | **0.018** | **0.015** | 0.722 | 0.230 |
| LYM | *r* | 0.299 | 0.211 | -0.177 | 0.196 | -0.195 | -0.050 | 0.060 |
|  | *P*-value | 0.082 | 0.223 | 0.308 | 0.259 | 0.262 | 0.774 | 0.733 |
| %LYM | *r* | **0.399^*^** | **0.438^**^** | 0.074 | **0.516^**^** | -0.212 | 0.044 | 0.251 |
|  | *P*-value | **0.018** | **0.008** | 0.671 | **0.002** | 0.221 | 0.803 | 0.147 |
| NEUT | *r* | -0.272 | **-0.398^*^** | -0.080 | **-0.435^**^** | 0.154 | -0.283 | -0.283 |
|  | *P*-value | 0.115 | **0.018** | 0.649 | **0.009** | 0.377 | 0.099 | 0.100 |
| %NEUT | *r* | -0.327 | **-0.503^**^** | 0.024 | **-0.549^**^** | 0.209 | -0.137 | -0.150 |
|  | *P*-value | 0.055 | **0.002** | 0.891 | **0.001** | 0.229 | 0.433 | 0.390 |
| PCT | *r* | -0.067 | -0.290 | 0.009 | -0.155 | -0.121 | -0.211 | 0.016 |
|  | *P*-value | 0.706 | 0.096 | 0.961 | 0.380 | 0.496 | 0.230 | 0.929 |
| Anxiety Score | *r* | 0.230 | 0.024 | -0.142 | 0.098 | -0.274 | -0.022 | 0.024 |
|  | *P*-value | 0.183 | 0.891 | 0.416 | 0.576 | 0.112 | 0.901 | 0.893 |

Note: FC, functional connectivity; R, right; L, left; *r*, Spearman correlation coefficient; C-reactive protein, CRP; alanine aminotransferase, ALT; aspartate aminotransferase, AST; albumin, ALB; globulin, GLOB; albumin/ globulin ratio, A/ G ratio; glomerular filtration rate, eGFR; creatine kinase, CK; white blood cell, WBC; lymphocytes, LYM; percentage of LYM, %LYM; neutrophil, NEUT; percentage of NEUT, %NEUT; procalcitonin, PCT. ^*^, Significant level *P* < 0.05; ^**^, Significant level *P* < 0.01.


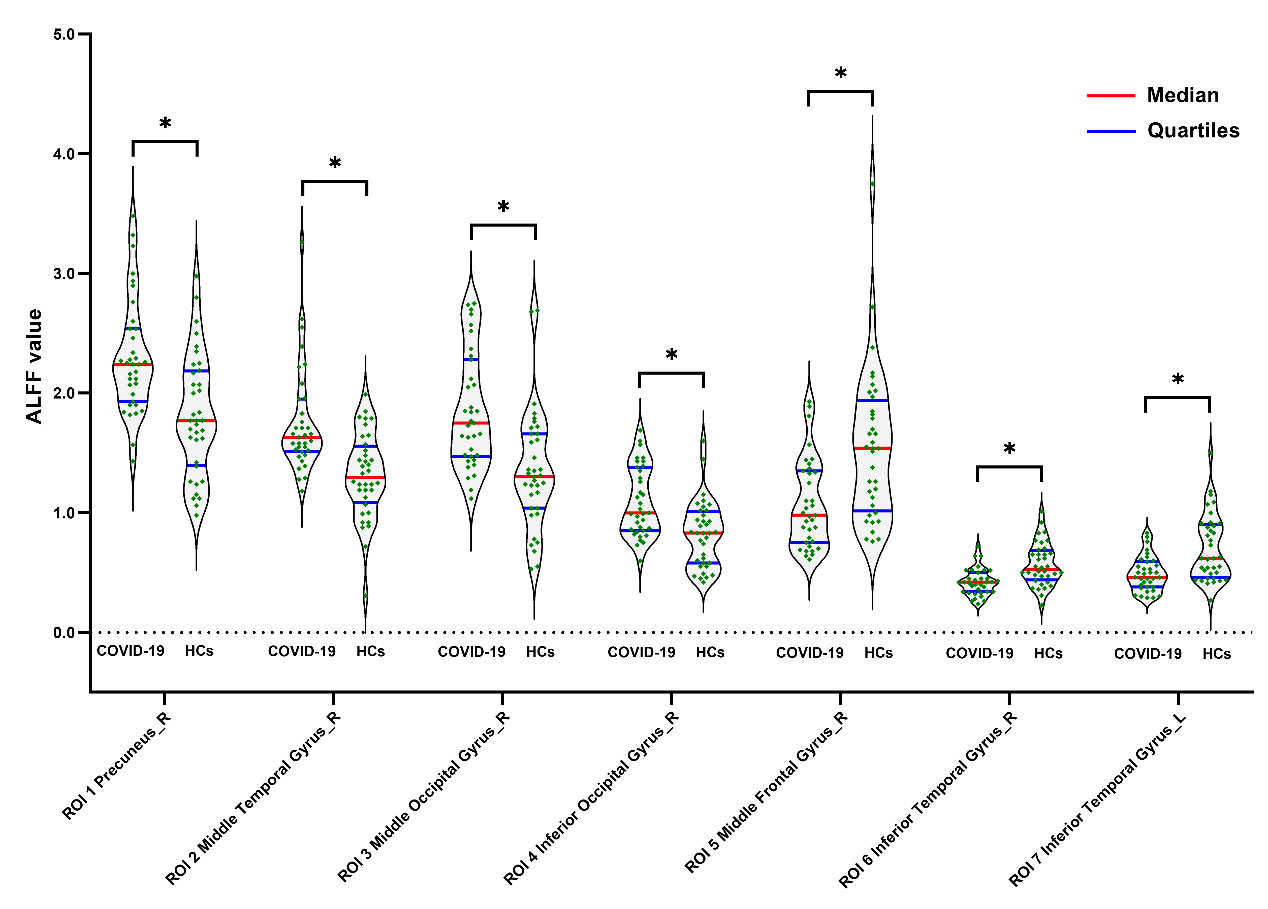


**Figure S1 Group differences in ALFF values**. The right precuneus, middle temporal gyrus, middle occipital gyrus, and inferior occipital gyrus exhibited increased ALFF (hyperactivity) in the recovered COVID-19 patients. The right middle frontal gyrus, and bilateral inferior temporal gyrus showed decreased ALFF (hypoactivity) (topological FDR corrected, *P* < 0.05). ALFF, amplitude of low-frequency fluctuations; HCs, healthy controls; ROI, region of interest; R, right; L, left; ^*^, Significant level *P* < 0.05.


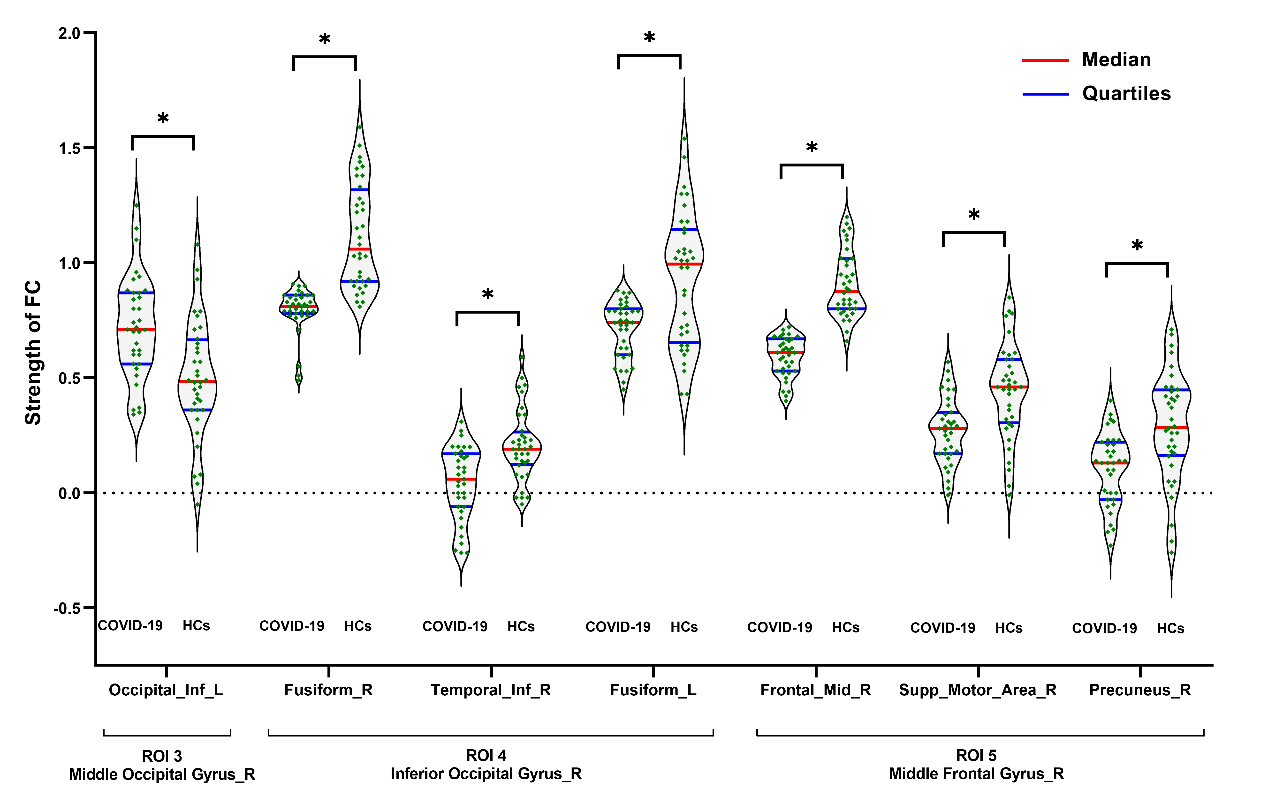


**Figure S2 Group differences in seed-based (ROI 3-5) FC values.** Relative to HC, recovered COVID-19 patients had significantly higher FC between the right middle occipital gyrus (ROI 3) and the left inferior occipital gyrus, lower FC between the right inferior occipital gyrus (ROI 4) and the right fusiform gyrus, inferior temporal gyrus, and left fusiform gyrus, lower FC between the right middle frontal gyrus (ROI 5) and the right frontal middle gyrus, supplementary motor area, and precuneus (topological FDR corrected, P < 0.05). FC, functional connectivity; HCs, healthy controls; ROI, region of interest; R, right; L, left; *, Significant level P < 0.05.
